# Validation of the Italian Version of the Rapid Geriatric Assessment in Community-Dwelling Older Adults

**DOI:** 10.3390/geriatrics10020038

**Published:** 2025-03-08

**Authors:** Carlotta Tacchino, Luca Carmisciano, Elena Page, Silvia Ottaviani, Luca Tagliafico, Alda Boccini, Alessio Signori, Chiara Giannotti, Alessio Nencioni, Fiammetta Monacelli

**Affiliations:** 1 Geriatrics Clinic, Department of Internal Medicine and Medical Specialties (DIMI), University of Genoa, 16132 Genoa, Italy; 2 IRCCS Ospedale Policlinico San Martino, 16131 Genoa, Italy; 3 Department of Health Science (DISSAL), University of Genoa, 16131 Genoa, Italy; 4 Department of Experimental Medicine (DIMES), University of Genoa, 16131 Genoa, Italy

**Keywords:** RGA, older adults, geriatric syndromes, screening

## Abstract

**Background/Objectives**: The Rapid Geriatric Assessment (RGA) is a tool designed to screen for frailty, sarcopenia, anorexia related to aging, and cognitive impairment. This study aimed to translate and validate the RGA for use among Italian community-dwelling older adults. **Methods**: This cross-cultural study involved 100 community-dwelling older adults randomly recruited through convenience sampling from general practitioner offices in Genoa (Italy), between January and June 2019. The RGA includes the Simple FRAIL Questionnaire Screening Tool, SARC-F Screening for Sarcopenia, Simplified Nutritional Assessment Questionnaire (SNAQ), and Rapid Cognitive Screening (RCS). These were validated against gold-standard tools: the Abbreviated Comprehensive Geriatric Assessment (aCGA) and Multidimensional Prognostic Index (MPI). Additional assessments included the Timed Up and Go (TUG) and Handgrip test. The validation process included forward–backward translation, synthesis, and consensus by independent reviewers. Psychometric properties, internal consistency (Cronbach alpha), and validity correlations were analyzed. **Results**: The RGA demonstrated satisfactory psychometric properties, with internal consistency (Cronbach alpha = 0.59) and significant validity correlations (RGA and aCGA, rho = 0.34, *p* = 0.001; RGA and MPI, rho = 0.49, *p* < 0.001). Discriminant validity was confirmed by significant correlations between specific subitems and reference measures: FRAIL with TUG (*p* < 0.05), SARC-F with Handgrip strength (*p* = 0.013), SNAQ with BMI, and RCS with MMSE (*p* < 0.001). **Conclusions**: The Italian version of the RGA is a reliable screening tool for geriatric syndromes in community-dwelling older adults. While it does not replace a CGA, the RGA may identify individuals who may benefit from further evaluation using a complete CGA.

## 1. Introduction

The demographic transition of the population has resulted in a shift in the population towards an increase in the number of older adults [1]. Although the process of aging is highly heterogeneous, increased life expectancy allows chronic diseases to develop, including geriatric syndromes, functional decline, disability, and frailty [2]. The prevalence of frailty among older age community dwellers is estimated to be 10%, highly depending on which frailty definition is used [3,4]. Frail older adults are the main users of medical and social services, with the current healthcare system unequipped to tackle such chronic and highly complex clinical management challenges [5].

The increased prevalence of older adults [6] represents a growing challenge for the sustainability of the healthcare system. The prevention of geriatric syndromes through early and multidimensional assessment in older adults seems to be the most effective option in reducing functional deterioration, disability, and frailty [7,8]. Systematic screening for frailty allows for risk stratification, which has been demonstrated to improve clinical outcomes, inform healthcare policymaking, and reduce the impact of older adults on healthcare systems [9]. The proper consideration of these concerns, together with an extensive screening for frailty and an early treatment of geriatric syndromes, results in better patient-centered care [10,11,12].

To promote fast screening of prevalent geriatric syndromes, Morley et al. [13] have developed Rapid Geriatric Assessment (RGA), a useful tool consisting of the «The Simple FRAIL Questionnaire Screening Tool», «SARC-F Screening for Sarcopenia», «Simplified Nutritional Assessment Questionnaire», and «Rapid Cognitive Screening». The RGA is a low-cost, easy-to-use, reliable, and time-saving tool that screens for frailty, sarcopenia, anorexia, and cognitive impairment.

The administration of the RGA does not require specialist expertise or advanced equipment, enabling its routine use in clinical practice. It is valuable for screening, identifying, and raising awareness of common geriatric syndromes, which are associated with increased mortality, functional decline, disability, reduced quality of life, and higher healthcare utilization [14].

Given this scientific background, the aim of the present study was to validate the Italian version of the RGA in a community-dwelling older population, in order to provide physicians and general practitioners (GPs) with an easy-to-use, time-saving, more accessible, and reliable tool for the early screening of frailty and other geriatric syndromes.

## 2. Materials and Methods

### 2.1. Study Population

One hundred (100) community-dwelling older adults from an urban area of Genoa, Liguria (Italy), were randomly enrolled from GP offices between January 2019 and June 2019, and were selected using convenience sampling from general practitioner offices in Genoa (Italy). The protocol of this study was approved by the local ethical committee, and all participants signed written informed consent. The inclusion criteria were patients aged 65 years or older, community dwellers, and Italian speakers, who had agreed to participate in the study and had read and signed the written informed consent. The procedures followed were in accordance with the ethical standards of the committee responsible for human experimentation (institutional and national) and with the 1975 Helsinki Declaration, revised in 2000.

Patients were excluded if no written informed consent was given, if they were a resident in a nursing home and/or any other semi-residential or residential setting, if they had severe multimorbidity (CIRS > 6) [15], advanced dementia (MMSE < 10/30) [16], end-stage chronic diseases (including cancer), major psychiatric diseases, severe sensorial deprivation, and/or if they were unable to read and/or appropriately understand the assessment tool.

### 2.2. Measurements

Demographic data, educational status, physical exercise habits, and patients’ medical history were collected at the baseline, and all patients underwent RGA, aCGA [17], and MPI [18] assessment.

The Abbreviated Comprehensive Geriatric Assessment (aCGA) included the following: Activities of Daily Living (ADL), the Barthel Index [19], and Instrumental Activities of Daily Living (IADL) [20] to assess functional status; the Mini Mental State Examination (MMSE) [16] to screen cognitive status; and the 5-item Geriatric Depression Scale (GDS 5-items) [21] to screen for depression.

Moreover, sarcopenia was screened through physical performance tests, such as the Timed Up and Go test (TUG) [22] and the Handgrip strength test (measured using a Handgrip dynamometer, Camry EH101(Sensun Weighing Apparatus Group Ltd, Guangdong, China), Units: Kg/libbers, Maximum capacity: 90 Kg/198 pounds, Power: 2X 1.5 V AAA batteries, Tolerance: ±0.5 Kg or 1 pound).

The Cumulative Illness Rating Scale (CIRS) [15] was used to assess multimorbidity, and the Gijón Scale was used to screen for social vulnerability [23].

The number of drugs, the number of falls, the presence of geriatric syndromes, and the presence of sensory deprivation were also collected and assessed at baseline.

### 2.3. Translation of the RGA Tool

An Italian geriatrician with advanced English skills and a native English speaker independently translated the original American English version of the RGA into Italian. The two professionals then discussed both translations; disagreements were resolved by consensus, and the final Italian version of the RGA tool was produced. Next, a back-translation was performed by a third independent professional English translator. The Italian version of the RGA (Supplementary S1) was provided for the validation process in a sample of a community-dwelling older adult population.

The pre-test for the semantic analysis of the final version for the clarity and comprehension of all items was conducted by 20 community-dwelling older age patients (10 women and 10 men) randomly enrolled in accordance with the inclusion criteria. These subsets of patients were not included in the validation assessment of the RGA tool.

### 2.4. Validation of the Italian Version of RGA

The Italian version of the RGA (Supplementary S1) was used in the validation process in a sample of 100 community-dwelling older adults. When assessing the psychometric properties of the tool, internal consistency (Cronbach’s alpha) and validity, as compared to the golden standards aCGA and MPI (rho; *p* value), were evaluated.

### 2.5. Statistical Analysis

Categorical variables were reported as counts and percentages, while continuous variables were described using means and standard deviations (SDs) and/or medians and interquartile ranges (IQR), based on their distribution. The standardized Cronbach’s alpha, based on correlations, was used to evaluate the internal consistency (reliability) of RGA. The results were reported as alpha, with its 95% confidence interval (95% CI), and the correlation of each item with the total score of the items was all standardized (r).

The concurrent, discriminant, convergent, and divergent validity criteria of the RGA were explored using Spearman’s rank correlation, with the results reported as rho coefficients and *p*-values.

A linear model was used to evaluate the association between tools, adjusting for age and gender. The results were reported as beta coefficients, standard errors (SE), and *p*-values. The normality of the fitted residuals was assessed using the Shapiro–Wilk normality test, with statistical significance reported where relevant.

When comparing the correlations between the tools and the general patient clinical characteristics, the false discovery rate method was applied to adjust the *p*-values for multiple comparisons. *p*-values below 0.05 were considered significant. R-software version 3.6.3 was used for all statistical analyses, together with the “psych” package version 1.9.12.

## 3. Results

### 3.1. Cohort Characteristics

The patients’ clinical characteristics are illustrated in Table 1.

The mean age of patients was 73 years (SD = 6.6), with a predominance of the female sex 71 (71%), a median multimorbidity of 3 (IQR = 2, 4), and a median drug use of 2 drugs (IQR = 1, 4).

### 3.2. Reliability and Validity of the RGA

Cronbach’s alpha of the RGA tool was 0.59 (95% CI 0.45, 0.72). Analysis of the correlation matrix showed significant correlations between the FRAIL, SARC-F, and RCS subitems (r ranging from 0.71 to 0.78), while a poorer correlation was observed for the SNAQ subitem (r = 0.40).

The RGA tool demonstrated satisfactory concurrent validity when correlated with the gold standards. Namely, a significative correlation between RGA and aCGA (rho = 0.34, *p*-value = 0.001) was found, as well as between RGA and MPI (rho = 0.49, *p*-value < 0.001). These associations remained statistically significant in linear models adjusted for age and sex (*p*-values of 0.027 for aCGA and 0.002 for MPI) (Table 2).

The RGA tool demonstrated strong discriminant validity, with a significant Spearman’s correlation between each RGA subitem and the corresponding reference tool. Namely, the FRAIL subitem was compared to the TUG test for frailty screening, the SARC-F tool was compared to the HG strength test to screen for sarcopenia, the SNAQ tool was compared to BMI for anorexia, and the RCS tool was compared to MMSE to screen for cognitive impairment (Table 3).

Table 4 illustrates the convergent and divergent validity between the RGA tool compared with aCGA and MPI, as well as patient clinical variables.

**Figure 1:**
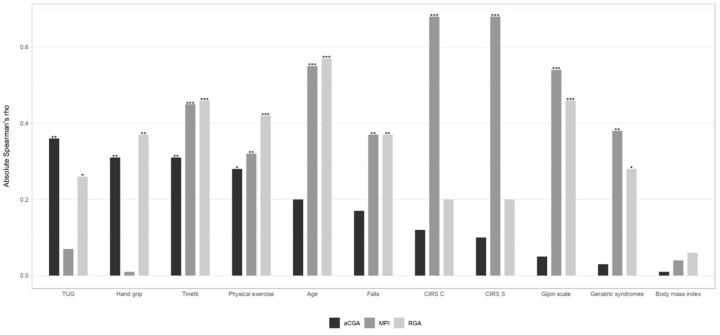
Spearman’s correlations between RGA tool, aCGA and MPI, respectively, and the patient’s clinical variables. * *p* < 0.05, ** *p* < 0.001, and *** *p* < 0.0001.

Age, the Gijón scale, number of falls, number of geriatric syndromes, Handgrip strength for sarcopenia screening, physical performance (TUG), and physical exercise showed stronger significant correlations with the RGA compared with aCGA and MPI (Figure 1). As expected, the MPI showed a stronger correlation with the CIRS than with the other scales, as it includes the CIRS as one of its components.

## 4. Discussion

The Comprehensive Geriatric Assessment (CGA) has long been regarded as the gold standard for geriatric assessment, demonstrating effectiveness in detecting problems that may otherwise go undetected until limitations arise or serious threats to independence occur [24,25]. However, while the comprehensive nature of the CGA is beneficial, especially in a hospital medical setting, it presents challenges for implementation in non-academic settings, particularly for clinicians with limited time and resources.

The Abbreviated CGA was designed by Overcash et al. to screen frail patients with cancer, identifying who would benefit from the entire CGA with a time-saving tool. Fifteen items were collected from the complete CGA to form the aCGA, evaluated through four assessment tools: ADL, IADL, MMSE, and GDS [26]. However, it is essential to clarify that the aCGA is not intended to replace the entire CGA, but to function as a screening tool to identify individuals who would benefit from undergoing the full CGA.

The Multidimensional Prognostic Index (MPI) is a comprehensive geriatric assessment tool derived from the CGA. The MPI utilizes a mathematical algorithm that incorporates data from eight distinct domains (ADL, IADL, SPMSQ, MNA, the Exton-Smith score, and CIRS), giving a single composite score ranging from 0 to 1, which has been shown to effectively predict mortality and other adverse outcomes in older adults (0 = no risk; 1 = higher risk of mortality) [27].

In the present study, we translated and validated the RGA tool into Italian for community-dwelling older adults, demonstrating its adequate psychometric properties for screening geriatric syndromes, including frailty [28].

Overall, the reliability analysis of the Italian version of the RGA tool showed a lower than expected but acceptable internal consistency. Poor consistency was observed in the reliability analysis of the SNAQ subitem. The reasons for this weaker association (*p*-value = 0.049), although speculative, may be linked to cultural and geographical differences in Italian nutritional habits and personal attitudes, which could impact anorexia in the Italian population. Malnutrition and anorexia often lead to weight loss; however, BMI is not always a sensitive indicator of malnutrition, particularly in older populations. BMI may fail to capture the nuances of anorexia or malnutrition that the SNAQ is designed to assess.

Furthermore, the Italian version of the RGA tool demonstrated adequate clinometric properties, supported by its concurrent validity compared to the gold standard aCGA and MPI, as well as discriminant validity across the RGA subitems (FRAIL, SARC-F, SNAQ, and RCS).

The Italian version of the RGA also showed strong convergent validity, correlating significantly with both the gold standards and other clinical variables.

In particular, the RGA tool had a significant correlation with age, number of falls, social vulnerability (Gijón scale), number of geriatric syndromes, physical performance, and physical activity, reinforcing its robust screening clinometric properties in a community setting. On the other hand, the low correlation between the RCA and the CIRS domains may reflect the different nature of the tools; the RCA domains focus on geriatric syndromes (such as frailty, sarcopenia, malnutrition, and cognitive decline), while the CIRS assesses multimorbidity, i.e., the coexistence of multiple chronic conditions.

The RGA offers several advantages in the assessment of older individuals. Its simplicity and rapid administration make it particularly useful in primary care settings, where time constraints often limit the feasibility of comprehensive geriatric assessments. Additionally, by identifying frailty, sarcopenia, anorexia, and cognitive impairment early, the RGA enables timely interventions that may help delay functional decline and reduce the healthcare burden. Its structured format also facilitates integration into electronic medical records, improving its accessibility for routine use in geriatric practice.

### 4.1. Sub-Items’ Internal Consistency

The positive relationship between the RGA subitem FRAIL and the Timed Up and Go test was statistically significant (*p* = 0.021), indicating a weak but meaningful correlation between the two tools in assessing frailty. The TUG test measures physical mobility and shows a strong association with specific components of the FRAIL scale, such as “sense of fatigue” and “ability to walk up one flight of stairs or walk one block”. However, since the FRAIL scale also assesses other factors like illness and weight loss, which are not directly related to physical performance, the overall correlation with TUG tends to be modest.

One of the key criteria for the diagnosis of sarcopenia according to the EWGSOP guidelines [29] is low muscle function (strength or performance). The Handgrip strength test provides an objective measure of muscle strength, while the SARC-F questionnaire helps assess subjective functional limitation, potentially diagnosing sarcopenia. The weak-to-moderate negative correlation between the SARC-F and HG strength test is statistically significant (*p*-value = 0.013); as the SARC-F scores increases, indicating greater perceived functional impairment, Handgrip strength tends to decrease, aligning with the expected decline in muscle strength associated with sarcopenia.

The cognitive domain assessed using RCS and MMSE showed a strong positive and statistically significant correlation (*p*-value < 0.001). Subjects who performed well on the RCS also tended to score higher on the MMSE, and vice versa. The strong correlation between the RCS and MMSE supports the use of RCS as a rapid screening tool for cognitive impairment, particularly in situations where a more comprehensive cognitive test, like the MMSE, may not be feasible, due to time constraints. However, the MMSE may still be necessary for a more detailed cognitive assessment.

### 4.2. Strengths and Limitations

A limitation of the study is its cross-sectional nature, which does not allow for the sensitivity to change of the RGA tool, and, similarly, the single measurement, which does not permit the evaluation of the stability of the translated tool in a test–retest setting or between different raters. Furthermore, the study’s results are based on a sample of 100 subjects from a single Italian city, which may limit the generalizability of the findings. In line with this, it seems of key importance to strengthen the results on a longitudinal basis, with prospective validation analyses, involving different populations, settings, and professional raters as well. Another limitation of this study is that alcohol and caffeine consumption were recorded as binary variables, without details on quantity or frequency. Additionally, while family history was assessed, data on certain conditions, such as tumor diseases and cardiovascular diseases, were not collected. Future studies should consider a more comprehensive assessment of these factors to provide a broader clinical context.

However, the strengths of the study rely on the validation of the Italian version of the RGA tool on a robust methodological basis in a “real-world” old age population, which allows for the availability of a reliable, affordable, time-saving, and easy-to-use tool, making it a meaningful screening tool for geriatric syndromes and frailty in community-dwelling older adults. Nevertheless, there is still only limited evidence to support the widespread adoption of multidimension-based tools, and the foundational factors that impact their accuracy and validity may be of key relevance for large-scale population screening [30,31].

The RGA is a brief screening tool requiring 4.5 min for administration by multidisciplinary team members, with a robust validation process in multiple countries and settings [32], and it can be implemented in electronic medical records, improving its practicability in busy routine GP offices.

## 5. Conclusions

This study successfully translated and validated the Italian version of the RGA tool for screening frailty in community-dwelling older adults, demonstrating its robust psychometric properties. The RGA exhibited strong correlations with established gold standards such as the aCGA and MPI, confirming its validity as a reliable screening instrument.

The availability of the Italian validated RGA tool could facilitate the widespread implementation of frailty and geriatric syndrome screening among frontline clinicians, especially in regions with aging populations, such as the older adults of Genoa. The RGA tool has significant potential to guide targeted interventions and cost-effective strategies, aiming to reduce the burden of geriatric syndromes, ultimately enhancing patient-centered care for older individuals. Future research should focus on the longitudinal validation of the RGA tool across diverse populations and clinical settings to further substantiate its reliability and utility as a versatile screening tool for frailty and its related conditions. However, it is important to articulate that RGA is not a replacement for the entire CGA, but rather a screening measure for those who would benefit from the entire CGA, to screen for potentially limiting health problems.

## Figures and Tables

**Table 1:** Patients’ clinical characteristics.

	Overall, N = 100
Age, Mean (SD)	73.35 (6.63)
Female, N (%)	71 (71.0)
BMI, Mean (SD)	24.43 (3.95)
Years of education, Median [IQR]	13.00 [10.00, 17.00]
Smoking	
Never smoked	44 (44.0)
Former smoker	48 (48.0)
Current smoker	8 (8.0)
Alcohol, N (%)	68 (68.0)
Caffeine, N (%)	77 (77.0)
CIRS-C, Median [IQR]	3.00 [2.00, 4.00]
CIRS-S, Median [IQR]	1.40 [1.30, 1.63]
Physical exercise	
3 to 7 times per week	13 (13.0)
1 or 2 times per week	47 (47.0)
Less than once a week	40 (40.0)
Family history (%)	
No	67 (67.0)
AD	23 (23.0)
Vascular	7 (7.0)
Parkinson’s disease	3 (3.0)
Vision impairment, N (%)	91 (92.9)

Abbreviation list: BMI: Body Mass Index; AD: Alzheimer’s disease; CIRS-C: Cumulative illness rating scale; CIRS-S: Cumulative illness rating scale severity.

**Table 2:** Analysis of the concurrent validity criterion of the RGA tool using Spearman’s correlation coefficients and linear regression between RGA and the gold standards, respectively, aCGA and MPI.

RGA Concurrent Validity	Spearman’s Correlation		Linear Regression ^§^	
	rho	*p*-Value	Beta (SE)	*p*-Value
aCGA	0.34	0.001 *	0.21 (0.10)	0.027 *
MPI	0.49	<0.001 *	2.40 (0.74)	0.002 *

Abbreviation list: aCGA: Abbreviated Comprehensive Geriatric Assessment; MPI: Multidimensional Prognostic Index; and ^§^ age and gender adjusted. Asterisks indicate statistical significance levels: * *p* < 0.05.

**Table 3:** Analysis of the discriminant validity of RGA subitems (FRAIL, SARC-F, SNAQ, and RCS) with the reference tool for the subitem, respectively, TUG, HG, BMI, and MMSE tool.

Domain	RGA Subitem	Reference Tool	Spearman’s Rho	*p*-Value
Frailty	FRAIL	TUG	0.23	0.021 *
Sarcopenia	SARC-F	HG	−0.25	0.013 *
Anorexia	SNAQ	BMI	0.19	0.049 *
Cognitive impairment	RCS	MMSE	0.67	<0.001 *

Abbreviation list: FRAIL: Simple FRAIL Questionnaire Screening Tool; SARC-F: Screen for Sarcopenia; SNAQ: Simplified Nutritional Assessment Questionnaire; RCS: Rapid Cognitive Screening; TUG: Timed Up and Go; HG: Handgrip dynamometer; BMI: Body Mass Index; and MMSE: Mini Mental State Examination. Asterisks indicate statistical significance levels: * *p* < 0.05.

**Table 4:** Spearman’s correlations between the RGA tool, aCGA, and MPI, respectively, and patient’s clinical variables.

	RGA		aCGA		MPI	
	rho	*p*	rho	*p*	rho	*p*
Age	0.57	<0.001	0.20	0.131	0.55	<0.001
Gijón scale	0.46	<0.001	0.05	0.783	0.54	<0.001
Falls	0.37	0.001	0.17	0.195	0.37	0.001
Geriatric syndromes	0.28	0.022	0.03	0.880	0.38	0.001
HG	−0.37	0.001	−0.31	0.008	0.01	0.953
TUG	0.26	0.036	0.36	0.001	0.07	0.682
Physical exercise	0.42	<0.001	0.28	0.021	0.32	0.005
Tinetti	−0.46	<0.001	−0.31	0.009	−0.45	<0.001
BMI	0.06	0.708	−0.01	0.960	0.04	0.849
CIRS-C	0.20	0.133	0.12	0.446	0.68	<0.001
CIRS-S	0.20	0.119	0.10	0.544	0.68	<0.001

Abbreviation list: BMI: Body Mass Index; HG: Handgrip dynamometer; TUG: Timed Up and Go; CIRS-C: Cumulative illness rating scale comorbidity index; CIRS-S: Cumulative illness rating scale severity index; RGA: Rapid Geriatric Assessment; aCGA: Abbreviated Comprehensive Geriatric Assessment; MPI: Multidimensional Prognostic Index.

## Data Availability

The raw data supporting the conclusions of this article will be made available by the authors upon request.

## Supplementary Materials

The following supporting information can be downloaded at: https://www.mdpi.com/article/10.3390/geriatrics10020038/s1. Supplementary S1: English and Italian translation of the RGA tool.
