# Supplementary material for: Validation of the Italian Version of the Rapid Geriatric Assessment in Community-Dwelling Older Adults
Source: Geriatrics (Basel). 2025 Mar 8;10(2):38. doi: 10.3390/geriatrics10020038 (PMC11932192; doi:10.3390/geriatrics10020038)
Supplement: Supplementary file 1 [file geriatrics-10-00038-s001.zip › geriatrics-3486082-supplementary.pdf]

Supplementary S1: English and Italian translation of the RGA tool

Simple FRAIL Questionnaire Screening Tool

Fatigue: Are you fatigued?

Resistance: Cannot walk up one flight of stairs?

Aerobic: Cannot walk one block?

Illnesses: Do you have more than 5 illnesses?

Loss of weight: Have you lost more than 5% of your weight in the last 6 months?

Scoring: 3 or greater = frailty; 1 or 2 = pre-frail

SARC-F

Strength: How much difficulty do you have in lifting and carrying 10 pounds?
None 0; Some 1; A lot or unable 2

Assistance in walking: How much difficulty do you have walking across a room?
None 0; Some 1; A lot, use aids, or unable 2

Rise from a chair: How much difficulty do you have transferring from a chair or bed?
None 0; Some 1; A lot or unable without help 2

Climb stairs: How much difficulty do you have climbing a flight of 10 stairs?
None 0; Some 1; A lot or unable 2

Falls: How many times have you fallen in the past year?
None 0; Less than 3 falls 1; 4 or more falls 2

Score $\geq$ 4 indicates Sarcopenia

SNAQ (Simplified Nutritional Assessment Questionnaire)

My appetite is:
a. very poor b. poor
c. average d. good
e. very good

When I eat
a. I feel full after eating only a few mouthfuls
b. I feel full after eating about a third of a meal
c. I feel full after eating over half a meal
d. I feel full after eating most of the meal
e. I hardly ever feel full

Food tastes
a. very bad
b. bad
c. average
d. good
e. very good

Normally I eat
a. less than one meal a day b. one meal a day
c. two meals a day
d. three meals a day
e. more than three meals a day

Scoring: a = 1, b = 2, c = 3, d = 4, e = 5.
Score $\leq$ 14 indicates significant risk of weight loss (>5%) in 6 months.

RCS (Rapid cognitive screen)

1 Please remember these five objects. I will ask you what they are later. Apple Pen Tie House Car
Please repeat the objects for me.

2. [Give patient pencil and the blank sheet with clock face.] This is a clock face. Please put in the hour markers and the time at ten minutes to eleven o'clock.
/2 (points) Hour markers okay
/2 (points) Time correct

3. What were the five objects I asked you to remember?
/1 (point) Apple
/1 (point) Pen
/1 (point) Tie
/1 (point) House
/1 (point) Car

4. I'm going to tell you a story. Please listen carefully because afterwards, I'm going to ask you about it.
"*Jill was a very successful stockbroker. She made a lot of money on the stock market. She then met Jack, a devastatingly handsome man. She married him and had three children. They lived in Chicago. She then stopped work and stayed at home to bring up her children. When they were teenagers, she went back to work. She and Jack lived happily ever after*".
What state did she live in?
/1 (point) Illinois

Scoring
8-10 .... Normal
6-7 .... Mild cognitive impairment
0-5 .... Dementia

Simple FRAIL Questionnaire Screening Tool

Fatica: è affaticato?

Resistenza: non riesce a fare un piano di scale?

Aerobica: non riesce a camminare per un isolato?

POLIPATOLOGIA: ha più di 5 malattie?

Significativa perdita di peso: ha perso più di 5% del peso negli ultimi 6 mesi?

Punteggio 3 o > = fragilità; 1 o 2 = pre-fragile

SARC-F

Salire le scale: quanta difficoltà ha nel salire 10 scalini?
Per nulla = 0; un po' = 1; molta difficoltà o incapace = 2

Assistenza: quanta difficoltà ha ad attraversare una stanza?
Per nulla = 0; Un po' = 1; molta difficoltà, utilizzo di ausili o incapace = 2

Alzarsi da una sedia: quanta difficoltà ha nel trasferirsi da una sedia al letto?
Per nulla = 0; Un po' = 1; molta difficoltà o incapace senza aiuto = 2

Cadute: quante volte è caduta nell'ultimo anno?
nessuna = 0; 1-3 cadute = 1; 4 o più cadute = 2

Forza: quanta difficoltà ha a sollevare e trasportare 4 kg?
nessuna= 0; Un po' = 1; molta difficoltà o incapace = 2

Punteggio ≥ 4 indice di Sarcopenia

SNAQ (Simplified Nutritional Assessment Questionnaire)

Il mio appetito è
a. molto ridotto
b. ridotto
c. nella norma
d.buono
e.ottimo

Il sapore del cibo è:
a. molto disgustoso
b. disgustoso
c. nella norma
d. buono
e. ottimo

Quando mangio:
a. mi sento sazio/a dopo pochi bocconi.
b. mi sento sazio/a dopo 1/3 del pasto.
c. mi sento sazio/a oltre la metà pasto.
d. mi sento sazio/a dopo aver mangiato la maggior parte del pasto.
e. non mi sento quasi mai sazio/a.

Normalmente io mangio:
a. meno di un pasto al giorno.
b. un pasto al giorno.
c. due pasti al giorno.
d. tre pasti al giorno
e. più di tre pasti al giorno.

Punteggio: a = 1, b = 2, c = 3, d = 4, e = 5.
Un punteggio ≤ 14 indica un rischio significativo di perdita di peso di almeno 5% entro 6 mesi.

RCS (Rapid cognitive screen)

1. Per favore, si ricordi questi 5 oggetti. Glieli richiederò più tardi.
(leggi ogni oggetto al paziente usando circa 1 sec di intervallo)
MELA SOLE LANA CASA GATTO

2. (Fornire la penna al paziente per il test dell'orologio)
Questo è un quadrante di un orologio. Per favore disponga le ore nel quadrante e quindi segni le ore le 11:10.
[2 pt/ lancette nella norma; 2 pt/tempo corretto]

- Quale sono i 5 oggetti che le ho chiesto di ricordare?
[1 pt/ognuno]

3. Le racconterò una storia. Per favore ascolti attentamente perché dopo le chiederò di ripetermela.

''Giulia fu un agente di cambio di grande successo. Fece un sacco di soldi per il mercato azionario. Poi incontrò Luca, un uomo incredibilmente bello. Si sposarono ed ebbero tre figli. Si trasferirono a vivere a Milano. Lei lasciò il lavoro e rimase a casa per allevare i suoi figli. Quando i figli furono adolescenti, ritornò al lavoro. Lei e Luca vissero felici e contenti.

In quale regione hanno vissuto? [1 pt]

PUNTEGGIO
8-10 .... Normale
6-7 .... Disturbo cognitivo lieve
0-5 .... Demenza

Abbreviated Comprehensive Geriatric Assessment (aCGA)—Revised for use in the older cancer patient. 132 J.A. Overcash et al. / Critical Reviews in Oncology/Hematology 54 (2005) 129–136

Patient Identifier____

GDS

To score the GDS (items 1-4) circle yes or no.

1. Do you feel that your life is empty? YES/NO
2. Do you feel happy most of the time? YES/NO
3. Do you often feel helpless? YES/NO
4. Do you feel pretty worthless the way you are now? YES/NO

ADL

To score the ADL (items 5-7) check which level of assistance applies.

5. Bathing (Sponge bath, tub bath or shower)

   ☐ Receive no assistance (gets into and out of tub by self if tub is the usual means of bathing)

   ☐ Receives assistance in bathing only one part of the body (such as the back or a leg)

   ☐ Receives assistance in bathing more than one part of the body (or not bathed)

6. Transfer

   ☐ Moves into and out of bed as well as into and out of chair without assistance (May use object such as cane or walker for support).

   ☐ Moves into or out of bed or chair with assistance.

   ☐ Doesn't get out of bed.

7. Continence

   ☐ Controls urination and bowel movement completely by self.

   ☐ Has occasional accidents

   ☐ Supervision helps keep control of urination or bowel movement or catheter is used or is incontinence.

IADL

To score the IADL (items 8-11) circle the number which reflects the ability.

8. Can you go shopping for groceries?

Without help		3
With some help	2	
Are you completely unable to do any shopping?	1	

9. Can you prepare your own meals?

Without help		3
With some help	2	
Are you completely unable to prepare any meals?	1	

10. Can you do your own housework?

Without help		3
With some help	2	
Are you completely unable to do any housework?	1	

11. Can you do your own laundry?

Without help		3
With some help	2	
Are you completely unable to do any laundry at all?	1	

MMSE
Score as indicated on each item.

12. Attention and Calculation

Begin with 100 and count backward by 7 (stop after 5 answers): 93,86, 79, 72, 65. Score one point for each correct answer. If the patient will not perform this task, ask the person to spell "WORLD" backwards (DLROW). Record the patient's spelling_______________________________________.
Score one point for each correctly placed letter. SCORE ______

13. Reading: Read and obey the following: Close your eyes (Show the patient the item on the attached paper).

CIRCLE THE SCORE 1 0

14. Writing: Write a sentence (on the attached paper).
CIRCLE THE SCORE 1 0

15. Copying: Copy the design of the intersecting pentagons.
CIRCLE THE SCORE 1 0

CLOSE YOUR EYES

WRITE A SENTENCE

COPY DESIGN
